# Validation of cross-sectional and longitudinal ComBat harmonization methods for magnetic resonance imaging data on a travelling subject cohort

**DOI:** 10.1016/j.ynirp.2022.100136

**Published:** 2022-10-06

**Authors:** Sophie Richter, Stefan Winzeck, Marta M. Correia, Evgenios N. Kornaropoulos, Anne Manktelow, Joanne Outtrim, Doris Chatfield, Jussi P. Posti, Olli Tenovuo, Guy B. Williams, David K. Menon, Virginia F.J. Newcombe

**Affiliations:** a Division of Anaesthesia, Department of Medicine, University of Cambridge, Cambridge, UK; b BioMedIA Group, Department of Computing, Imperial College London, London, UK; c MRC Cognition and Brain Sciences Unit, University of Cambridge, Cambridge, UK; d Diagnostic Radiology, Lund University, Lund, Sweden; e Turku Brain Injury Center, Turku University Hospital & University of Turku, Turku, Finland; f Department of Neurosurgery, Turku University Hospital, Turku, Finland; g Wolfson Brain Imaging Centre, Department of Clinical Neurosciences, University of Cambridge, Cambridge, UK

**Keywords:** Up to 6): magnetic resonance imaging, Harmonization, ComBat, Travelling subject, Diffusion tensor imaging

## Abstract

**Background:**

The growth in multi-center neuroimaging studies generated a need for methods that mitigate the differences in hardware and acquisition protocols across sites i.e., scanner effects. ComBat harmonization methods have shown promise but have not yet been tested on all the data types commonly studied with magnetic resonance imaging (MRI). This study aimed to validate neuroCombat, longCombat and gamCombat on both structural and diffusion metrics in both cross-sectional and longitudinal data.

**Methods:**

We used a travelling subject design whereby 73 healthy volunteers contributed 161 scans across two sites and four machines using one T1 and five diffusion MRI protocols. Scanner was defined as a composite of site, machine and protocol. A common pipeline extracted two structural metrics (volumes and cortical thickness) and two diffusion tensor imaging metrics (mean diffusivity and fractional anisotropy) for seven regions of interest including gray and (except for cortical thickness) white matter regions.

**Results:**

Structural data exhibited no significant scanner effect and therefore did not benefit from harmonization in our particular cohort. Indeed, attempting harmonization obscured the true biological effect for some regions of interest. Diffusion data contained marked scanner effects and was successfully harmonized by all methods, resulting in smaller scanner effects and better detection of true biological effects. LongCombat less effectively reduced the scanner effect for cross-sectional white matter data but had a slightly lower probability of incorrectly finding group differences in simulations, compared to neuroCombat and gamCombat. False positive rates for all methods and all metrics did not significantly exceed 5%.

**Conclusions:**

Statistical harmonization of structural data is not always necessary and harmonization in the absence of a scanner effect may be harmful. Harmonization of diffusion MRI data is highly recommended with neuroCombat, longCombat and gamCombat performing well in cross-sectional and longitudinal settings.

## Highlights

• ComBat harmonization was validated on travelling subjects using cross-sectional and longitudinal data.
• Structural data did not exhibit scanner effects and was harmed by harmonization.
• ComBat harmonization successfully removes scanner effects in diffusion data.
• False positive rates for ComBat harmonization methods do not significantly exceed 5%.

## Graphical abstract

**Figure:**
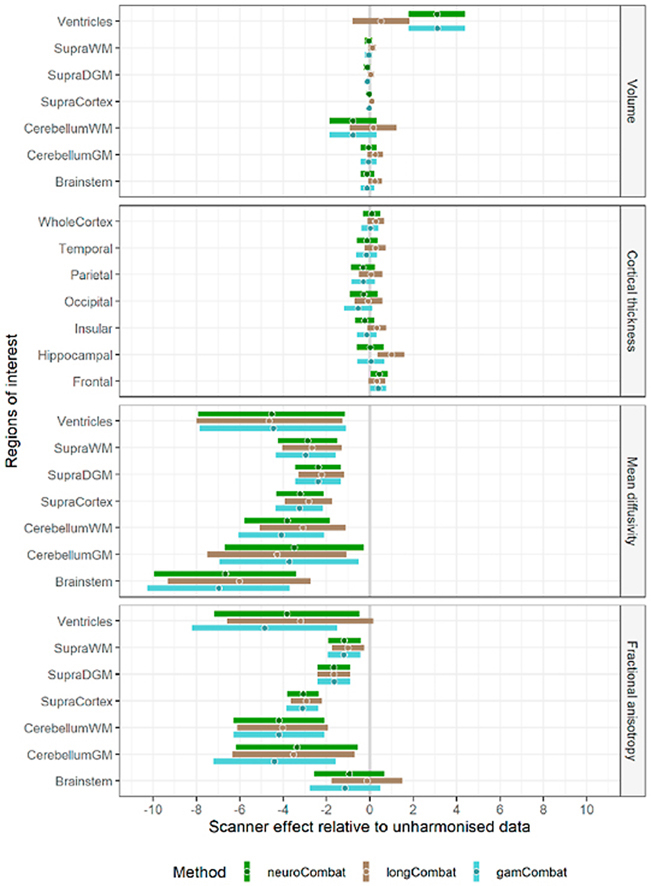

## 1 Introduction

Recent years have seen a growth in collaborative neuro-imaging studies. Whilst these allow for recruitment of larger and more representative populations, they also introduce the challenge of accounting for different scanner hardware and acquisition settings used at each study site. This difference between scanners, or “scanner effect”, can be of similar magnitude as the difference between patients and controls (Pinto et al., 2020). Thus without correction for scanner effects (i.e., harmonization), the gain in statistical power from larger patient numbers would be cancelled out by the increase in noise.

Prospective studies (e.g., CENTER-TBI (Maas et al., 2015), TRACK-TBI (Yue et al., 2013), IMAGEN (Schumann et al., 2010)) have the opportunity to reduce scanner-effects by agreeing a priori on uniform acquisition protocols across sites, although hardware differences between sites persist. Retrospective or evolving collaborations however, such as the Enhancing NeuroImaging Genetics through Meta-Analysis (ENIGMA) consortium and the Alzheimer's Disease Neuroimaging Initiative (ADNI) database, have to also consolidate acquisition protocols that vary across sites or needed to be updated over time.

To capitalize on the wealth of existing imaging data and continue fruitful collaborations, the imaging community needs robust harmonization methods that have been validated on all the data types and imaging metrics that they are intended to be used on.

A variety of harmonization methods have been developed to minimize the unwanted scanner effect but preserve the biological variability (Pinto et al., 2020). These include methods to harmonize the images themselves and those applied to the extracted features (e.g., regional cortical thickness). The drawbacks of harmonizing images themselves include the need to share actual images across sites (data sharing, anonymization and data volume challenges), the greater demand on computational resources and often a priori requirements for study design (e.g., participants to be matched in age and gender across sites, or protocols to be consistent across sites). For these reasons, harmonizing already extracted imaging features is often more practicable.

A popular method for the harmonization of extracted features is ComBat (“combating batch effects when combining batches”), which was originally developed for genomic data (Johnson et al., 2007) and has then been adapted to neuroimages (R package “neuroCombat”) (Fortin et al., 2017, 2018). NeuroCombat adjusts the values of the extracted imaging features, so that the whole cohort can be treated as if all images had been obtained from the same scanner. Expected values are estimated using a linear model with biological variables (such as age and sex) as well as additive and multiplicative scanner effects as predictors (Fortin et al., 2018). Empirical Bayes is used to better estimate the model parameters for small sample sizes (Fortin et al., 2018).

NeuroCombat has been shown to increase statistical power superior to other feature-harmonization methods in cross-sectional population data: when associating age with cortical thickness (Fortin et al., 2018) or with diffusion metrics (fractional anisotropy (FA) and mean diffusivity (MD)) (Fortin et al., 2017), and when comparing patients with controls with regards to cortical thickness, cortical surface area and volumes of sub-cortical nuclei (Radua et al., 2020). Other examples of applications to cross-sectional data include the use for positron emission tomography (Orlhac et al., 2018) and functional MRI (Yu et al., 2018). More recently, Pomponio et al. modified neuroCombat to allow for non-linear covariate effects by using generalized additive models (from now on referred to as gamCombat) and applied their method to study volumetric changes across the human lifespan (Pomponio et al., 2020). Beer et al. adapted ComBat specifically to longitudinal data (R package longCombat) and showed that longCombat was more powerful for detecting a change in cortical thickness over time, compared to neuroCombat (Beer et al., 2020).

The aforementioned studies are limited by the absence of a “ground truth”. How much of the scanner effect a harmonization method has removed and how much of the true biological effect it has preserved can only be known when using a “travelling subject “cohort. A “travelling subject” is a participant that is scanned on multiple scanners within a short timeframe, to allow for these scanners to be compared. One such cohort, where the same 20 subjects were scanned on three different scanners, has been used to assess the performance of neuroCombat on cortical thickness, cortical and total sub-cortical volume (Maikusa et al., 2021). The authors found that neuroCombat did remove some but not all of the scanner effect (Maikusa et al., 2021). This study was limited though by the fact that scans of the same subject were up to 14 months apart so that true cortical loss during the study period cannot be excluded. In addition, the performance on longitudinal and/or diffusion data was not assessed.

In summary, ComBat harmonization is a promising technique but not all variants have been validated in cross-sectional cohorts for individual tissue volumetric data and none have been validated in longitudinal cohorts for volumetric or diffusion data.

The present study therefore aims to answer the following question, using a travelling subject cohort: Given that MRI data can be cross-sectional or longitudinal, and can include structural or diffusion metrics - do all of these data types benefit from harmonization and, if yes, which ComBat harmonization method (neuroCombat, longCombat or gamCombat) is best for each data type?

## 2 Methods

### 2.1 Participants

Participants were healthy controls imaged between August 22, 2006 and December 17, 2019 at either the Wolfson Brain Imaging Center, Cambridge (UK) or the Turku University Hospital (Finland). Ethical approval was obtained from the Cambridgeshire Local Research Ethics Committee (LREC 97/290), the Norfolk Research Committee (REC EE/0395), the NHS Health Research Authority (14/SC/1370) and the Ethical Committee of the Hospital District of South-West Finland (decision 68/180/2011). Written consent was obtained for all participants.

This study assessed the performance of ComBat harmonization methods on both cross-sectional and longitudinal data, necessitating three participant cohorts with different eligibility criteria. The cross-sectional data approach compares the scan-rescan variability in subjects who had two scans less than 180 days apart on the same scanner (within-scanner cohort) with the scan-rescan variability in subjects who had two scans less than 180 days apart on different scanners (across-scanner cohort). We refer to this cohort as cross-sectional to differentiate it from the longitudinal cohort although even in this cohort each subject had more than one scan (a pre-requisite for calculating a ground truth and for implementing the some of the harmonization algorithms tested here). The longitudinal data approach uses subjects who had a reference scan followed by two follow-up scans more than 365 days later, one on the same and one on a different scanner to the reference scan (longitudinal cohort). Scanner was defined as a composite of site, manufacturer model and acquisition settings.

### 2.2 Image acquisition

Images were acquired at both study sites on 3T MRI scanners (Trio, Verio and Prisma models of Siemens Medical Solutions, Erlangen, Germany) and included structural and/or diffusion MRI.

Structural images were acquired as T1-weighted images using magnetization-prepared rapid acquisition with gradient echo (MPRAGE). Both sites used TE = 2.98 ms, TR = 2300 ms, TI = 900 ms, flip angle = 9 deg, matrix size = 256 × 240 x 176 1 mm isotropic voxels with sagittal slices.

Diffusion MRI protocols are summarized in Table 1. Note that for the multi-shell scans both the posterior to anterior and the anterior to posterior phase encoding directions were collected, to correct for phase-encoding direction induced distortions.

**Table 1:** Summary of acquisition protocols used for diffusion tensor imaging.

Protocol	A	B	C	D	E
**Site**	Turku	Cambridge	Cambridge	Cambridge	Cambridge
**Shells**	Single	Single	Single	Multi	Multi
**Bands**	Single	Single	Single	Single	Multi
**Directions**	64	32	63	12	98
**b-values (s/mm**^**2**^**)**	1000	1000	1000	350, 650, 1000, 1300, 1600	300, 1000, 2000
**B = 0**	1	1	1	1	5
**Voxel size (mm**^**2**^**)**	2 × 2 × 2	2 × 2 × 2	2 × 2 × 2	2 × 2 × 2	1.75 × 1.75 × 1.75
**TE (ms)**	106	91	106	119	75
**TR (s)**	11.7	9.8	11.7	12.3	2.433
**Field of view (mm)**	192	256	192	192	192
**Matrix size**	96 × 96	128 × 128	96 × 96	96 × 96	110 × 110
**Slices**	77	75	63	63	76

### 2.3 Image processing

All images were processed on a common pipeline (Winzeck, 2021) to extract two structural metrics (volume and mean cortical thickness) and two diffusion metrics (means of fractional anisotropy (FA) and mean diffusivity (MD)) in seven regions of interest (ROIs): ventricles, cortical gray matter, supra-tentorial white matter, supra-tentorial deep gray matter, cerebellar gray matter, cerebellar white matter, brainstem; or for cortical thickness: frontal, insular, parietal, occipital, temporal, hippocampal and whole cortex. In brief, images were neck cropped and corrected for scanner field inhomogeneities. Diffusion tensor images were corrected for noise, Gibbs ringing, eddy current and motion artefacts and field inhomogeneities (Manjón et al., 2013; Veraart et al., 2016a, 2016b; Jeurissen et al., 2014; Andersson and Sotiropoulos, 2016). FSL (Jenkinson et al., 2012) was used for weighted-least squares estimation of diffusion tensors to calculated FA and MD maps. T1w images were parcellated into ROIs using MALP-EM (Ledig et al., 2015) and rigidly co-registered to diffusion tensor imaging (DTI) space to extract mean FA and MD values for each ROI. Cortical thickness was computed using diffeomorphic registration-based cortical thickness (DiReCT) estimation via nipype's built-in interface for the ANTS KellyKapowskialgorithm (Das et al., 2009). All processed images were visually inspected for quality assurance.

### 2.4 Statistical analysis

All statistical analysis was performed in R (version 4.1.1) (R Core Team. R, 2021). P-values were adjusted for multiple comparison's within each column of each table using Holm's method.

#### 2 .4.1 Harmonizing data

Harmonization was performed using the R packages neuroCombat_1.0.13 (“https://github.com/Jfortin1/neuroCombat_Rpackage”) (Fortin et al., 2017, 2018), longCombat_0.0.0.90000 (“https://github.com/jcbeer/longCombat”) (Beer et al., 2020) and neuroHarmonize, which we refer to as gamCombat, (“https://github.com/rpomponio/neuroHarmonize”). (Pomponio et al., 2020) Harmonization was performed separately for each of the metrics (volume, cortical thickness, MD and FA) and for each of the two analysis approaches. Covariates used in the harmonization included “age”, “sex”, “intracranial volume” and “time since the first scan”. For the estimation of the false positive rate (FPR) “group” was also included (a randomly assigned label of either A or B, see section 2.4.4). For neuroCombat we used default settings i.e., a parametric prior for the main analysis. We also conducted sensitivity analyses using a non-parametric prior (argument parametric = FALSE) as well as fitting a non-bayesian location-shift model (argument eb = FALSE). For longCombat default settings were used with the formula and ranef arguments corresponding to the respective mixed model used in subsequent analysis. For cross-sectional data we estimated a subject-specific intercept (ranef = (1|subject)); for longitudinal data we estimated either a subject-specific intercept only (ranef = (1 | subject)) or a subject-specific intercept and slope over time (ranef = (1 + time | subject)) formethods “longCombat_i” and “longCombat_i + s” respectively. For gamCombat we used default settings and specified a non-linear effect for the covariate age (smooth_terms = age).

#### 2.4.2 Comparing performance of different harmonization methods

**Fig. 1:**
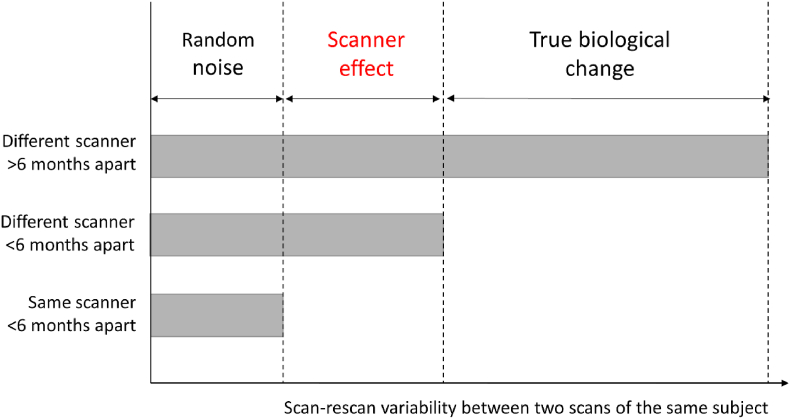
**Components of scan-rescan variability within the same subject.** Random noise is comprised of physiological (e.g., subject related), thermal noise (e.g., acquisition related) and statistical noise (e.g., image processing related). Scanner effect is the part of the scan-rescan difference amenable to harmonization and is caused by differences in the hardware and/or acquisition settings between the first and the second scan. True biological change is the measurement of interest in longitudinal studies e.g., the reduction in cortical volume or fractional anisotropy over time.

For the cross-sectional data, the scan-rescan variability was measured using the coefficient of variation expressed as a percentage (CoV = standard deviation/mean*100) for each scan pair in each person. The mean CoV in the within-scanner cohort provides a measure of random noise not amenable to harmonization. This random noise is caused by a combination of physiological noise (e.g., hydration status of the subject), thermal noise (e.g., acquisition related) and statistical noise (e.g., stochastic steps in the image processing pipeline) (Fig. 1). Here, a CoV of 2% can be interpreted, for example, as follows: when measuring the ventricular volume of the same subject repeatedly on the same scanner, the standard deviation across repeat scans will be 2% percent of the mean ventricular volume. The mean CoV in the across-scanner cohort provides a measure of random noise plus scanner effect (Fig. 1). Therefore, if the mean CoV in the across-scanner cohort is significantly larger than the mean CoV in the within-scanner cohort, then there is a significant scanner effect. For example, if the CoV in the within-scanner cohort is estimated at 2% and the CoV in the across-scanner cohort at 3%, then the extra 1% of noise can be ascribed to the scanner effect. The CoV of each of the across-scanner cohorts (unharmonized, neuroCombat-harmonized,longCombat-harmonized and gamCombat-harmonized) was compared with the within-scanner cohort using a *t*-test. P-values were adjusted for multiple comparisons using Holm's method and considered significant if < 0.05. For significant scanner effects the magnitude of this effect was estimated using Cohen's d (Cohen, 1992).

In the longitudinal cohort each subject had one initial reference scan and two follow-up scans, one on the same scanner as the reference scan (within-scanner follow-up) and one on a different scanner (across-scanner follow up) at least 365 days after the reference scan. The two follow up scans were done within approximately 6 months of each other: median (range) for structural and diffusion cohorts were 6.6 (0.0–53.3) and 6.3 (0.0–53.3) months. To correct for any differences in the follow-up interval, the annual rate of change (e.g., volume loss per year) was calculated between the reference scan and each of the follow-up scans. This allowed for the calculation of what the imaging metric for the across-scanner follow-up would have been, had that scan been done on the same day as the within-scanner follow-up. A sample calculation for volume would be:

$$Volume_{adjusted}=Volume_{reference}+\left(Time_{within}*Rate_{across}\right)$$

where Volume_reference_ is the volume on the initial reference scan, Volume_adjusted_ is the volume from the follow-up scan on a different scanner, Time_within_ is the time between the reference scan and the within-scanner follow-up and Rate_across_ is the rate of volume change per unit time measured between the reference scan and the across-scanner follow-up. The discrepancy between the within-scanner and adjusted across-scanner follow-ups was considered to be caused by differences in hardware and acquisition settings, i.e., the scanner effect. We quantified the scanner effect for each subject using the percentage CoV. One would expect the CoV to be large in unharmonized data and reduced in harmonized data. To compare the CoV between harmonization methods taking account of repeated measures within subjects, we fitted a linear mixed model with CoV as the independent variable, method as a fixed effect and subject as a random effect. A harmonization method was considered to have had a statistically significant effect, if the 95% confidence interval for its coefficient did not cross 0.

#### 2.4.3 Assessing whether harmonization obscures true biological effects

The correlation between the biological effect observed after harmonization and the true biological effect (ground truth) was assessed with the intra-class correlation coefficient. A value of 1 would indicate that the entire biological effect was detectable after harmonization, a value of zero would indicate that the entire biological effect was obscured after harmonization. The biological effect in question was the annual rate of change in the imaging parameter between the initial reference scan and the follow-up scan (approximately 3–5 years later), e.g., volume loss in supratentorial white matter. The ground truth was the annualized rate of changed measured when rescanning the subject on the same scanner as the original reference scan.

#### 2.4.4 Comparing the false positive rate of different harmonization methods

Each subject was randomly assigned to either group A or group B. Mixed models were fitted to test for a difference between group A and B with respect to the intercept (e.g., Does group A have higher volumes than group B?) and the slope (e.g., Does group A experience greater loss of white matter per unit time than group B?). Models also controlled for age and sex (as fixed effects) and repeated within-subject measurements (random effect). Any significant difference (p < 0.05) between the groups was considered a false positive. The FPR was calculated by repeating the random group assignment 1000 times and counting the number of unadjusted p-values <0.05. This simulation was performed for each of the seven ROIs for the original and all harmonized datasets (neuroCombat, longCombat and gamCombat). The Friedman test, a non-parametric equivalent to the repeated measures ANOVA, was used to compare the FPRs of the different harmonization methods with each other. To decide whether each individual FPR for each method, metric and data type was acceptable, we tested whether the FPR was significantly greater than the widely accepted 5% threshold using a one-sample one-sided Wilcoxon test.

### 2.5 Data and code availability

The R code for the statistical analysis is publicly available at https://github.com/DrSophieRichter/Validate_ComBat. Upon request de-identified imaging data can be made available to individual research groups, by submitting a formal project outline and signing data sharing agreements with each site. The authors are also open to applying future harmonization algorithms on this dataset to support development and assessment of novel methods (please contact the corresponding author if interested).

## 3 Results

This study included 161 scans from 73 participants. Cohort characteristics are summarized in Table 2. The median scan-rescan interval was three weeks or less, and more than three years in the cross-sectional and longitudinal cohorts respectively. All cohorts contained approximately equal numbers of men and women, with an age range representing most of the adult lifespan.

**Table 2:** Characteristics of cohorts included in the analysis. The numbers between structural and diffusion tensor images differ because the definition of “scanner” included acquisition characteristics. E.g., a subject may have been scanned twice on the same machine using identical settings for structural image acquisition but different settings for diffusion image acquisition on the two occasions.

Cohort	Cross-sectional within scanner	Cross-sectional across scanner	Longitudinal cohort
Inclusion criteria	Each subject had 2 scans on the same scanner less than 180 days apart	Each subject had 2 scans on two different scanners less than 180 days apart	Each subject had one reference scan and 2 follow-up scans more than 365 days later. One follow-up scan was on the same, the other on a different scanner to the reference scan.
Used in	Cross-sectional approach	Cross-sectional approach	Longitudinal approach
Structural images			
Subjects	62	23	17
Scans	124	46	51
Scanners	4	5	3
Time between scans, median (range)	21.5 (2.0–180.0) days	7.0 (0.0–160.0) days	Within: 5.0 (1.6–9.4) years Across: 5.2 (1.8–9.5) years
Age, median (range)	33 (19–84)	38 (26–59)	39 (24–62)
Male sex, count (%)	32 (52%)	11 (48%)	10 (59%)
Diffusion tensor images			
Subjects	39	32	14
Scans	78	64	42
Scanners	5	9	6
Time between scans, median (range)	20.0 (2.0–180.0) days	13.5 (0.0–160.0) days	Within: 3.2 (1.4–9.2) years Across: 3.3 (1.8–12.8) years
Age, median (range)	33 (20–84)	34 (19–59)	38 (25–64)
Male sex, count (%)	19 (49%)	17 (53%)	8 (57%)

### 3.1 Performance of different harmonization methods applied to cross-sectional data

The performance of the three harmonization methods (neuroCombat, longCombat and gamCombat) applied to cross-sectional data is summarized in Table 3. The CoV in the within-scanner cohort, i.e., random noise, ranged between 0.8 and 5.5% depending on the metric and ROI investigated.

**Table 3:**
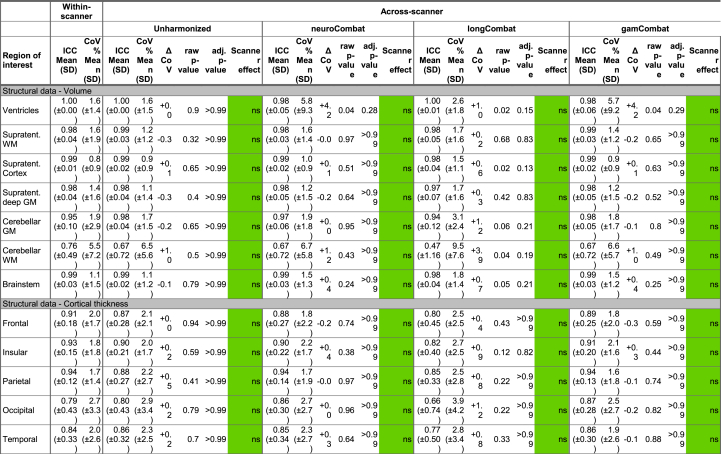

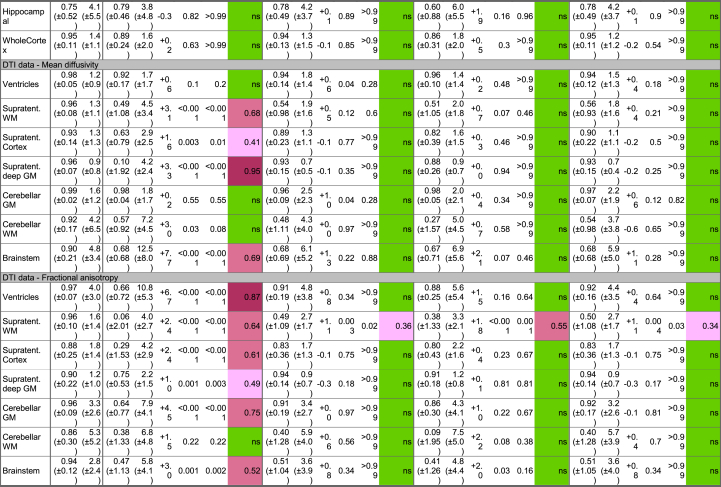
**Scanner effect before and after harmonization.** Healthy subjects were scanned twice less than 180 days apart on either the same scanner (Within-scanner) or on two different scanners (Across-scanner). ICC = intra-class correlation coefficient. CoV = Coefficient of variation. This can be interpreted, for example for line 1, as follows: when measuring the ventricular volume of the same subject repeatedly on the same scanner, the standard deviation across repeat scans will be 1.6% (±1.4) of the mean ventricular volume. ΔCoV is the across-scanner CoV minus the within-scanner CoV i.e., a measure of how much variation is added by using a different scanner for the second scan. The within-scanner CoV and across-scanner CoV were compared with a *t*-test. Raw p-value and adj. p-value show the p-values before and after adjustment for multiple comparisons using Holm's method. We considered the use of different scanners to have a significant effect if adj. p-value < 0.05. Where this was the case, the magnitude of this scanner effect is calculated as Cohen's d with the color coding of purple/pink/rose for large/medium/small effects with the thresholds of 0.8, 0.5 and 0.2 respectively. Non-significant scanner effects (ns) are coloured green. Supratent. = Supratentorial, WM = white matter, GM = gray matter, DTI = diffusion tensor imaging.

For structural data the CoV in the across-scanner cohort did not differ significantly from that in the within-scanner cohort, suggesting that the use of different scanners did not introduce any additional noise which could have been targeted by harmonization. Attempting nonetheless to harmonize structural data had no significant effect.

For DTI metrics the CoV in the across-scanner cohort differed significantly from that in the within-scanner cohort for most ROIs, indicating small-large scanner effects. NeuroCombat, longCombat and gamCombat, successfully removed all significant scanner effect, with one exception: The scanner effect apparent in the supratentorial white matter could only be reduced but not completely removed (Cohen's d 0.36, 0.55 and 0.34 after neuroCombat, longCombat and gamCombat harmonization respectively).Sensitivity analyses showed that the version of neuroCombat presented in Table 3, which is using a parametric prior, performs better than alternative implementations with a non-parametric prior or a non-bayesian approach (supplemental Table 1).

### 3.2 Performance of different harmonization methods applied to longitudinal data

**Fig. 2:**
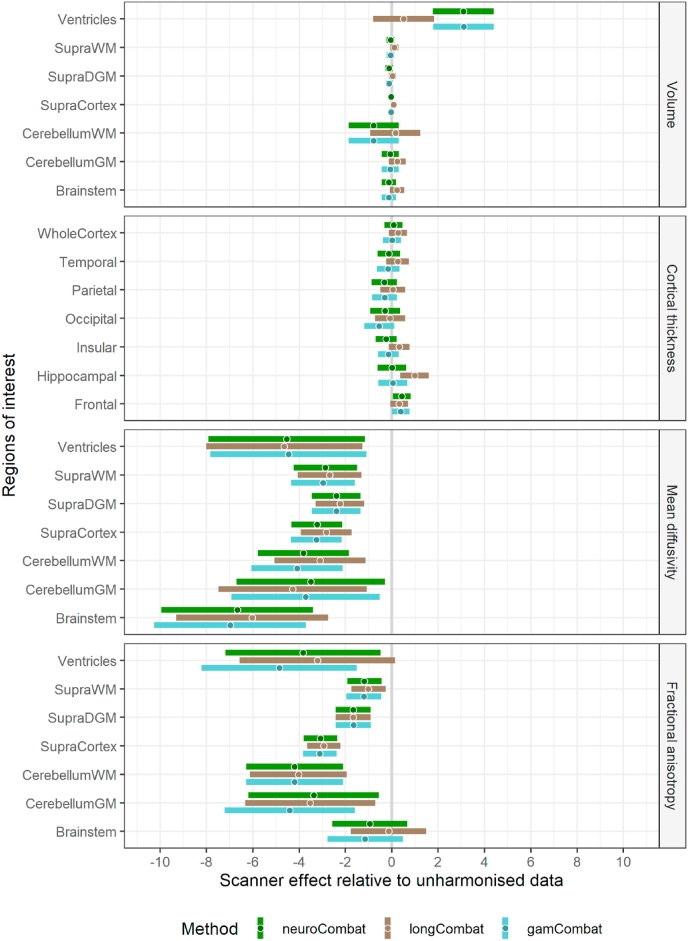
Scanner effect relative to unharmonized data. Subjects received an initial reference scan and two follow-up scans at least one year later, one on the same and one on a different scanner. The difference between the two follow-up scans is considered to be due to differences in the hardware and acquisition settings, i.e., due to the scanner effect. The scanner effect for each subject was expressed as the percentage coefficient of variation (CoV). Here the CoV in harmonized data is shown relative to the CoV of unharmonized data, i.e., if the CoV of unharmonized data is x%, a value of −1 on the forest plot means harmonization has reduced the CoV to x-1%. Thus, points to the left of the vertical gray line (denoting zero scanner effect) indicate a reduction in scanner effect, points to the right an increase in scanner effect. A harmonization method reached statistical significance if its bars (95% confidence intervals) did not cross the gray line. The harmonization methods assessed were: neuroCombat, longCombat (witha subject-specific intercept) and gamCombat (with age as the non-linear covariate). Abbreviations in the names of regions of interest are: Supra = supra-tentorial, WM = white matter, (D)GM = (deep) gray matter.

The ability of the three harmonization methods (neuroCombat, longCombat, and gamCombat) to reduce the scanner effect in longitudinal data is illustrated in Fig. 2. Points to the left of the vertical gray line (denoting zero scanner effect) indicate a reduction in scanner effect, points to the right an increase in scanner effect. A harmonization method reached statistical significance if its bars (95% confidence intervals) did not cross the gray line.

For structural data none of the harmonization methods significantly altered the scanner effect in any ROI, with two exceptions. NeuroCombat and gamCombat applied to ventricular volumes inadvertently increased the scanner effect by 3 percentage points compared to unharmonized data. LongCombat methods applied to hippocampal cortical thickness increased the scanner effect by 1 percentage point.

For diffusion data all harmonization methods significantly reduced the scanner effect in all ROIs, with two exceptions. LongCombat methods just failed to reach significance for FA in the ventricles (where measuring FA is arguably meaningless) as did all methods in the brainstem.

A sensitivity analysis using different settings for neuroCombat and longCombat yielded similar results (supplemental figures 1 and 2).

### 3.3 Assessment of whether harmonization obscures true biological effects

The ability of different harmonization methods to detect the true biological change over time when the follow up scan is performed on a different scanner to the initial reference scan, is summarized in Table 4.

**Table 4:**
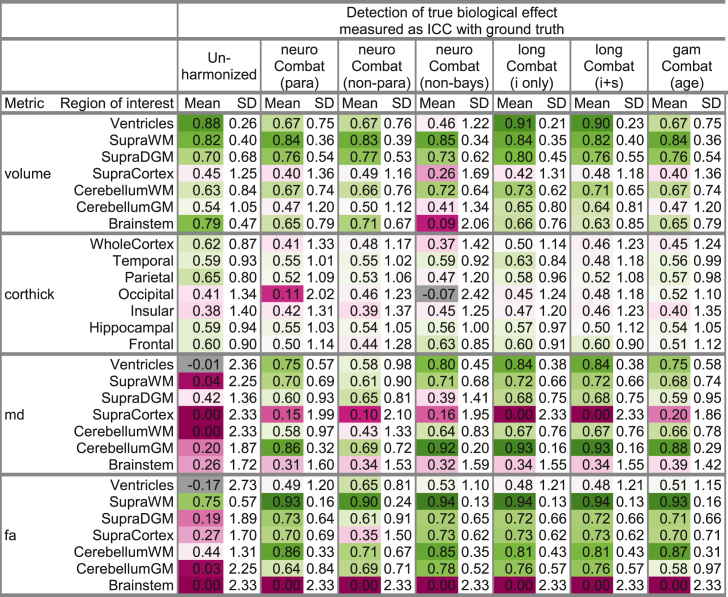
Detection of true biological effect before and after harmonization. The biological effect in question was the annual rate of change in the imaging parameter between the initial reference scan and the follow-up scan (approximately 3–5 years later), e.g., volume loss in supratentorial white matter. The ground truth was the annualized rate of changed measured when rescanning the subject on the same scanner as the original reference scan. The ICC (intra-class correlation coefficient) measures the agreement between the biological effect observed when rescanning the subject on a different scanner and the ground truth. Good agreement (ICC >0.5) is color-coded in green, poor agreement is color-coded in purple. Abbreviations: Supra = supra-tentorial, WM = white matter, (D)GM = (deep) gray matter, para/non-para = parametric/non-parametric prior, non-bays = non-bayesian implementation (location-shift model), i only = subject-specific intercept only, i + s = subject-specific intercept and slope, age = non-linearity assumed for covariate age.

For structural data, the observed biological effect correlated well with the ground truth even in unharmonized data (most intra-class correlation coefficients >0.5). This correlation was not enhanced by harmonization. Indeed, the biological effect was slightly obscured by harmonization. This was especially pronounced when using the non-bayesian neuroCombat implementation (most regions of interest), when harmonizing whole cortex cortical thickness (all harmonization methods) and when harmonizing ventricular volumes (all but longCombat methods).

For DTI metrics the biological effect was largely lost in unharmonized data (most intra-class correlation coefficients <0.5) but restored by harmonization, irrespective of the method used.

### 3.4 False positive rate using different harmonization methods

**Fig. 3:**
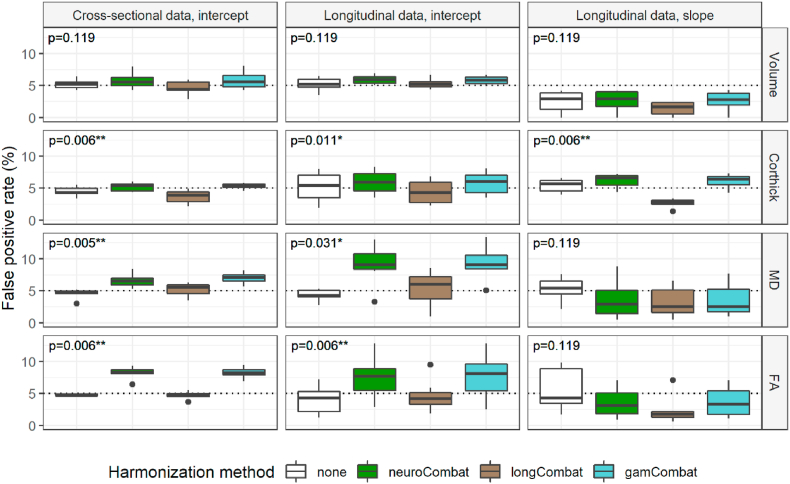
False positive rates (FPR) compared across harmonization methods. Subjects were randomly assigned to group A or B and this was repeated 1000 times. The false positive rate equates to the percentage of times a significant difference was found between groups. By chance, a 5% FPR is expected (dotted line). The columns indicate the type of data used and the research question asked. “Cross-sectional data” refers to the dataset where the scan-rescan interval of each subject was less than 180 days. “Longitudinal data” refers to the dataset in which the scan-rescan interval of each subject was greater than 365 days. The data was used to either look at a difference in intercept (e.g., “Does group A have larger volumes than group B″) or a difference in slope (e.g., “Does group A experience greater white matter volume loss per unit time than group B″). Rows refer to the metrics assessed: volume, cortical thickness (Corthick), mean diffusivity (MD) or fractional anisotropy (FA). Each boxplot shows the median and interquartile range of the seven regions of interest (see methods). The number of false positives generated by the four methods (no harmonization, neuroCombat, longCombat and gamCombat) have been compared using the Friedman test with p-values stated in the top left corner of each panel. P-values have been adjusted for multiple comparisons using Holm's method and an asterisk indicates a p-value < 0.05 after adjustment. To assess whether any individual box represented a false positive rate exceeding the widely acceptable 5% threshold, a one-sample one-sided Wilcoxon test was used. After correction for multiple comparisons, none of the false positive rates significantly exceeded the 5% threshold.

Median false positive rates ranged around the 5% mark expected by chance and remained below 10% for all harmonization methods (Fig. 3). Indeed, after adjustment for multiple comparisons, none of the false positive rates significantly exceeded the widely accepted 5% threshold.

Testing for a difference in intercept (e.g., “Does group A have larger volumes than group B”), the neuroCombat- or gamCombat-harmonized data generated more false positives than unharmonized or longCombat harmonized data. After adjustment for multiple comparisons this difference remained significant for all metrics except volume, both when using cross-sectional data and when using longitudinal data.

Testing for a difference in slope (e.g., “Does group A experience greater white matter loss per unit time than group B?“), longCombat-harmonized data tended to produce fewer false positives than unharmonized, neuroCombat- or gamCombat-harmonized data, although after correction for multiple comparisons this only remained significant for cortical thickness.

## 4 Discussion

This is the first comprehensive validation of cross-sectional and longitudinal ComBat harmonization methods for neuroimaging data on travelling subjects (i.e., the same subject scanned on multiple scanners). We demonstrate overall robust performance of all methods, neuroCombat, longCombat and gamCombat, with regards to power and false positive rates, for harmonizing cross-sectional and longitudinal, structural and diffusion data.

To the authors‘ knowledge this is the largest study of ComBat harmonization using travelling subjects to date (and only the second) (Maikusa et al., 2021), the first to include diffusion data and the first to include longitudinal travelling subject data.

The scan-rescan variability observed in our study, both within and across scanners, compares well with those previously reported for volumes (Wittens et al., 2021; De Guio et al., 2016; Jovicich et al., 2009; Kruggel et al., 2010; Yang et al., 2016; Fujita et al., 2019; Vavasour et al., 2019; Deprez et al., 2018), cortical thickness (Fujita et al., 2019; Kecskemeti et al., 2021; Mcguire et al., 2017) and diffusion metrics (Acheson et al., 2017; Grech‐Sollars et al., 2015; Kamagata et al., 2015; Liu et al., 2014; Palacios et al., 2017; Prohl et al., 2019; Shahim et al., 2017; Veenith et al., 2013; Zhou et al., 2018).

First, we assessed the effect of harmonizing structural data. Interestingly, we did not detect any significant scanner effect in the unharmonized data, suggesting that harmonization of structural data in our cohort was not necessary. Most imaging studies would not include travelling subjects, so would not know whether a scanner effect is present and might proceed to harmonization “just in case”. We therefore applied the ComBat harmonization methods to our structural data nonetheless to see what the effect would be in a dataset without significant scanner differences.

Importantly, we detected increased scanner effects after harmonizing ventricular volumes and hippocampal cortical thickness in longitudinal data. The effect on hippocampal cortical thickness is very small and might be a false positive, considering that 95% confidence intervals are not corrected for multiple comparisons. The effect on ventricular volume however is larger and apparent in both, the cross-sectional and the longitudinal analysis, especially when using neuroCombat or gamCombat (although it did not survive testing for multiple comparisons in the cross-sectional analysis). It is possible that the scanner characteristics exert different effects on cerebrospinal fluid (CSF) compared to tissue and that CSF volumes need to be harmonized separately from tissue volumes. This however is not possible unless more than one CSF ROI is available as ComBat harmonization techniques require more than one ROI. Since the scanner effect was undetectable for unharmonized ventricular volumes, we would consider it safest to use unharmonized data when studying ventricular volumes.

For structural metrics (volumes and cortical thickness) in other ROIs we found neither an increase nor a decreasein the scanner effect after harmonizing the data. Worryingly, we found that harmonization methods obscured some of the true biological effect in structural data. This contradicts previous findings which suggested a gain in power by using neuroCombat (Fortin et al., 2018; Radua et al., 2020; Maikusa et al., 2021) or longCombat (Beer et al., 2020). There are two possible explanations for this, the first being a difference in sample size. With our study being a travelling subject cohort study, it is necessarily smaller than previous studies (using hundreds to thousands of subjects)(Fortin et al., 2018; Radua et al., 2020; Beer et al., 2020) and may have been underpowered to detect a small scanner effect. Secondly, all our structural images were acquired using the same sequence type (MPRAGE) and acquisition parameters, on models of the same vendor (Siemens), and were processed on a common pipeline at the same site. Previous studies combined different sequences, vendors and pipeline variations. This may explain the absence of a significant scanner effect in our structural data.and may have shifted the cost-benefit equation of harmonization so that the cost of reducing some true biological variance outweighed the benefit of reducing the scanner-related variance.

For diffusion metrics (MD and FA) our results suggest a great reduction of scanner effect after harmonization. This agrees with a previous study applying neuroCombat to a cross-sectional dataset (Fortin et al., 2017). We extend these findings by showing that neuroCombat also works on longitudinal diffusion data, and that longCombat works on cross-sectional and longitudinal diffusion data. Both methods yielded almost identical results on both types of data. They differed in their performance on supra-tentorial white matter in cross-sectional data, where neuroCombat, which was developed for cross-sectional data, was superior. Whilst in longitudinal data the two methods performed differently in the ventricles and the brainstem, we believe that these differences could be disregarded: the change in FA and MD in the ventricles is not usually considered of biological interest. Furthermore, there are lots of possible technical confounders when measuring FA in the brainstem such as disproportionate motion introduced by vascular pulsations and a slight mismatch in the neck cropping level between the diffusion images and the co-registered structural template.

The false positive rates in our study did not significantly exceed the 5% threshold and were thus acceptable for all harmonization methods. The size of the FPRs observed in our study agree with those reported by Beer et al. who applied neuroCombat and longCombat to cortical thickness data (Beer et al., 2020). Consistent with their simulation study on null features i.e., testing for a difference where there is none, neuroCombat had slightly higher FPRs than longCombat (Beer et al., 2020).

The strengths of our study include the travelling subject cohort design which means that there is an absolute ground truth to compare the effects of harmonization to. The ground truth here is the data obtained from the same subject being imaged twice on the same scanner; the comparator is the harmonized data obtained from the same subject being scanned on two different scanners. To our knowledge it is the first travelling subject study on the topic with truly longitudinal data. Second, the age and sex mix of this study is a good representation of the general adult population, an important consideration since scan-rescan variability varies with age (Jovicich et al., 2009). Second, we assessed performance on a range of metrics and ROIs in both diffusion and structural data to provide a comprehensive overview. Third, two complementary analysis approaches, on cross-sectional and longitudinal data, provided similar results which lends weight to our conclusions.

Weaknesses of this analysis include the aforementioned limited sample size and inter-scanner difference which may have prohibited the detection of small benefits when harmonizing structural data. Whilst repeat scans of the same subject were performed within a few weeks, ideally this time interval would be even shorter to completely eliminate any true biological change between repeat scans. Furthermore, scanner drift was not controlled for in the longitudinal data which may have affected the within and across-scanner follow-up in different ways. However there were no major scanner updates during the study period and the effect of scanner-drift has previously been shown to be negligible compared to the scanner effect (Jovicich et al., 2009). Finally, the performance of longCombat must be interpreted in context. Most cross-sectional studies do not have more than one scan per subject, so cannot apply longCombat. Most longitudinal studies have either across-scanner or within-scanner data available for each participant, but rarely both, so longCombat may perform less well than in our study. The other tested ComBat algorithms however are agnostic to repeated measures per subject, so their performance estimates should generalize well to other study designs.

## 5 Conclusion

We conclude that harmonization is optional for structural data acquired with uniform acquisition settings on machines of the same vendor, and in some instances bestavoided. Harmonization however is highly recommended for diffusion data. We showed that neuroCombat, longCombat and gamCombat are powerful methods for harmonizing diffusion data in both the cross-sectional and longitudinal settings, with neuroCombat being preferable for cross-sectional data (better performance on white matter) and longCombat preferable for longitudinal data (due to lower false positive rates).

## Funding

Data collection was supported by the 10.13039/100011102European Union 7th Framework Programme, EC grant 602150 and FP7-270259-TBIcare), with additional funding from OneMind, NeuroTrauma Sciences, Integra Neurosciences, and 10.13039/501100000781European Research Council 10.13039/501100007601Horizon 2020, EC grant 757173). Infrastructure was provided by the 10.13039/501100000272NIHR Cambridge Biomedical Research Centre and the NIHR Cambridge Clinical Research Facility, which is a partnership between Cambridge University Hospitals NHS (National Health Service) Foundation Trust and the 10.13039/501100000735University of Cambridge, funded by the 10.13039/501100000272NIHR. Individuals were supported by a 10.13039/100010269Wellcome Trust PhD Fellowship (222213/Z/20/Z) (Dr Richter); by an 10.13039/501100000272NIHR Senior Investigator Award (Dr Menon); by the 10.13039/501100002341Academy of Finland (17379) (Dr Posti) and Maire Taponen Foundation (Dr Posti); and by the 10.13039/501100000691Academy of Medical Sciences/The 10.13039/501100000724Health Foundation (UK) (Dr Newcombe). The views expressed are those of the authors and not necessarily those of the NIHR or the Department of Health and Social Care. The funders had no role in the study design; in the collection, analysis and interpretation of data; in the writing of the report; and in the decision to submit the article for publication.

## Author contributions

**Sophie Richter:** Conceptualization, Methodology, Formal analysis, Data curation, Writing- Original Draft, Visualization **Stefan Winzeck:** Methodology, Software, Data Curation, Writing – Review & Editing **Marta M. Correia:** Methodology, Supervision, Writing – Review & Editing **Evgenios N. Kornaropoulos:** Software, Writing – Review & Editing **Anne Manktelow**: Investigation, Writing – Review & Editing **Joanne Outtrim**: Investigation, Writing – Review & Editing **Dot Chatfield:** Investigation, Writing – Review & Editing **Jussi Posti:** Investigation, Writing – Review & Editing **Olli Tenovuo:** Funding acquisition, Project administration, Writing – Review & Editing **Guy B. Williams:** Resources, Writing – Review & Editing **David K. Menon:** Funding acquisition, Project administration, Supervision, Writing – Review & Editing **Virginia F. J. Newcombe:** Methodology, Supervision, Writing – Review & Editing.

## Declaration of competing interest

The authors declare the following financial interests/personal relationships which may be considered as potential competing interests: David Menon received personal fees from Lantmannen AB, 10.13039/100004330GlaxoSmithKline plc, Calico Life Sciences LLC, PresSura Neuro, Integra Neurosciences, and NeuroTrauma Sciences, LLC; grants from 10.13039/100004330GlaxoSmithKline plc; and a shared 10.13039/100000002National Institutes of Health grant from Gryphon Collaborators on a grant application outside the presented work. Virginia Newcombe holds grants from F. Hoffman–La Roche Ltd and received personal fees from Neurodiem Honorarium for a talk put into the 10.13039/501100000735University of Cambridge research fund outside the presented work.

## Data Availability

Data will be made available on request.
