# Supplementary material for: Validation of cross-sectional and longitudinal ComBat harmonization methods for magnetic resonance imaging data on a travelling subject cohort
Source: Neuroimage Rep. 2022 Oct 6;2(4):100136. doi: 10.1016/j.ynirp.2022.100136 (PMC9726680; doi:10.1016/j.ynirp.2022.100136)
Supplement: Multimedia component 1 [file mmc1.docx]

Supplement to “Validation of cross-sectional and longitudinal ComBat harmonization methods for magnetic resonance imaging data on a travelling subject cohort”

# Contents

1. Supplemental table 1. Scanner effect before and after harmonization for neuroCombat not using a parametric prior
2. Supplemental figure 1. Scanner effect before and after harmonization in longitudinal data.
3. Supplemental figure 2. Scanner effect relative to unharmonized data for variants of longCombat and neuroCombat methods

|  | **Within-scanner** | | **Across-scanner** | | | | | | | | | | | | | | | | | |
| --- | --- | --- | --- | --- | --- | --- | --- | --- | --- | --- | --- | --- | --- | --- | --- | --- | --- | --- | --- | --- |
|  |  | | **Unharmonized** | | | | | | **neuroCombat non-parametric prior** | | | | | | **neuroCombat non-baysian** | | | | | |
| **Region of interest** | **ICC Mean (SD)** | **CoV % Mean (SD)** | **ICC Mean (SD)** | **CoV % Mean (SD)** | **Δ CoV** | **raw p-value** | **adj. p-value** | **Scanner effect** | **ICC Mean (SD)** | **CoV % Mean (SD)** | **Δ CoV** | **raw p-value** | **adj. p-value** | **Scanner effect** | **ICC Mean (SD)** | **CoV % Mean (SD)** | **Δ CoV** | **raw p-value** | **adj. p-value** | **Scanner effect** |
| Structural data - Volume | | | | | | | | | | | | | | | | | | | | |
| Ventricles | 1.00 (±0.00) | 1.6 (±1.4) | 1.00 (±0.00) | 1.6 (±1.5) | +0.0 | 0.9 | >0.99 | ns | 0.98 (±0.04) | 5.8 (±8.0) | +4.3 | 0.02 | 0.13 | ns | 0.97 (±0.07) | 6.7 (±8.2) | +5.2 | 0.007 | 0.05 | 0.35 |
| Supratent. WM | 0.98 (±0.04) | 1.6 (±1.9) | 0.99 (±0.03) | 1.2 (±1.2) | -0.3 | 0.32 | >0.99 | ns | 0.98 (±0.04) | 1.7 (±1.3) | +0.1 | 0.82 | >0.99 | ns | 0.98 (±0.05) | 1.9 (±1.5) | +0.3 | 0.48 | >0.99 | ns |
| Supratent. Cortex | 0.99 (±0.01) | 0.8 (±0.9) | 0.99 (±0.02) | 0.9 (±0.9) | +0.1 | 0.65 | >0.99 | ns | 0.99 (±0.02) | 0.9 (±0.9) | +0.0 | 0.84 | >0.99 | ns | 0.99 (±0.03) | 1.1 (±1.0) | +0.3 | 0.22 | >0.99 | ns |
| Supratent. deep GM | 0.98 (±0.04) | 1.4 (±1.6) | 0.98 (±0.04) | 1.1 (±1.4) | -0.3 | 0.4 | >0.99 | ns | 0.98 (±0.04) | 1.2 (±1.4) | -0.2 | 0.61 | >0.99 | ns | 0.98 (±0.05) | 1.4 (±1.5) | -0.0 | 0.9 | >0.99 | ns |
| Cerebellar GM | 0.95 (±0.10) | 1.9 (±2.9) | 0.98 (±0.04) | 1.7 (±1.5) | -0.2 | 0.65 | >0.99 | ns | 0.97 (±0.07) | 2.2 (±2.1) | +0.3 | 0.65 | >0.99 | ns | 0.96 (±0.08) | 2.1 (±2.1) | +0.2 | 0.71 | >0.99 | ns |
| Cerebellar WM | 0.76 (±0.49) | 5.5 (±7.2) | 0.67 (±0.72) | 6.5 (±5.6) | +1.0 | 0.5 | >0.99 | ns | 0.70 (±0.66) | 6.8 (±5.6) | +1.2 | 0.41 | >0.99 | ns | 0.68 (±0.71) | 7.1 (±5.7) | +1.6 | 0.3 | >0.99 | ns |
| Brainstem | 0.99 (±0.03) | 1.1 (±1.5) | 0.99 (±0.02) | 1.1 (±1.2) | -0.1 | 0.79 | >0.99 | ns | 0.99 (±0.03) | 1.4 (±1.3) | +0.3 | 0.36 | >0.99 | ns | 0.98 (±0.04) | 1.6 (±1.4) | +0.5 | 0.16 | 0.94 | ns |
| Structural data - Cortical thickness | | | | | | | | | | | | | | | | | | | | |
| Frontal | 0.91 (±0.18) | 2.0 (±1.7) | 0.87 (±0.28) | 2.1 (±2.1) | +0.0 | 0.94 | >0.99 | ns | 0.88 (±0.27) | 2.0 (±2.2) | -0.1 | 0.91 | >0.99 | ns | 0.87 (±0.28) | 1.8 (±2.3) | -0.2 | 0.68 | >0.99 | ns |
| Insular | 0.93 (±0.15) | 1.8 (±1.8) | 0.90 (±0.21) | 2.0 (±1.7) | +0.2 | 0.59 | >0.99 | ns | 0.87 (±0.28) | 2.5 (±2.1) | +0.7 | 0.17 | >0.99 | ns | 0.90 (±0.22) | 2.0 (±1.7) | +0.2 | 0.58 | >0.99 | ns |
| Parietal | 0.94 (±0.12) | 1.7 (±1.4) | 0.88 (±0.27) | 2.2 (±2.7) | +0.5 | 0.41 | >0.99 | ns | 0.93 (±0.16) | 2.0 (±1.9) | +0.3 | 0.49 | >0.99 | ns | 0.93 (±0.15) | 1.9 (±1.8) | +0.2 | 0.66 | >0.99 | ns |
| Occipital | 0.79 (±0.43) | 2.7 (±3.3) | 0.80 (±0.43) | 2.9 (±3.4) | +0.2 | 0.79 | >0.99 | ns | 0.86 (±0.30) | 2.6 (±2.7) | -0.1 | 0.9 | >0.99 | ns | 0.88 (±0.26) | 2.5 (±2.8) | -0.2 | 0.8 | >0.99 | ns |
| Temporal | 0.84 (±0.33) | 2.0 (±2.6) | 0.86 (±0.32) | 2.3 (±2.5) | +0.2 | 0.7 | >0.99 | ns | 0.84 (±0.34) | 2.4 (±2.7) | +0.4 | 0.53 | >0.99 | ns | 0.84 (±0.36) | 2.4 (±2.8) | +0.3 | 0.62 | >0.99 | ns |
| Hippocampal | 0.75 (±0.52) | 4.1 (±5.5) | 0.79 (±0.46) | 3.8 (±4.8) | -0.3 | 0.82 | >0.99 | ns | 0.77 (±0.51) | 4.2 (±4.3) | +0.1 | 0.92 | >0.99 | ns | 0.77 (±0.51) | 4.7 (±3.5) | +0.6 | 0.59 | >0.99 | ns |
| WholeCortex | 0.95 (±0.11) | 1.4 (±1.1) | 0.89 (±0.24) | 1.6 (±2.0) | +0.2 | 0.63 | >0.99 | ns | 0.94 (±0.14) | 1.3 (±1.5) | -0.1 | 0.87 | >0.99 | ns | 0.94 (±0.12) | 1.3 (±1.4) | -0.1 | 0.81 | >0.99 | ns |
| DTI data - Mean diffusivity | | | | | | | | | | | | | | | | | | | | |
| Ventricles | 0.98 (±0.05) | 1.2 (±0.9) | 0.92 (±0.17) | 1.7 (±1.7) | +0.6 | 0.1 | 0.2 | ns | 0.78 (±0.47) | 2.8 (±3.7) | +1.6 | 0.02 | 0.14 | ns | 0.93 (±0.16) | 2.0 (±1.6) | +0.8 | 0.01 | 0.06 | ns |
| Supratent. WM | 0.96 (±0.08) | 1.3 (±1.1) | 0.49 (±1.08) | 4.5 (±3.4) | +3.1 | <0.001 | <0.001 | 0.68 | 0.62 (±0.80) | 1.7 (±1.3) | +0.3 | 0.26 | 0.77 | ns | 0.50 (±1.08) | 1.9 (±1.7) | +0.6 | 0.09 | 0.43 | ns |
| Supratent. Cortex | 0.93 (±0.14) | 1.3 (±1.3) | 0.63 (±0.79) | 2.9 (±2.5) | +1.6 | 0.003 | 0.01 | 0.41 | 0.81 (±0.40) | 1.7 (±1.4) | +0.3 | 0.28 | 0.77 | ns | 0.88 (±0.25) | 1.3 (±1.2) | +0.0 | 0.99 | >0.99 | ns |
| Supratent. deep GM | 0.96 (±0.07) | 0.9 (±0.8) | 0.10 (±1.92) | 4.2 (±2.4) | +3.3 | <0.001 | <0.001 | 0.95 | 0.82 (±0.38) | 1.3 (±0.8) | +0.4 | 0.04 | 0.19 | ns | 0.92 (±0.17) | 0.7 (±0.4) | -0.2 | 0.32 | >0.99 | ns |
| Cerebellar GM | 0.99 (±0.02) | 1.6 (±1.2) | 0.98 (±0.04) | 1.8 (±1.7) | +0.2 | 0.55 | 0.55 | ns | 0.93 (±0.14) | 3.5 (±2.8) | +1.9 | 0.001 | 0.007 | 0.43 | 0.91 (±0.19) | 3.6 (±3.5) | +2.0 | 0.004 | 0.03 | 0.34 |
| Cerebellar WM | 0.92 (±0.17) | 4.2 (±6.5) | 0.57 (±0.92) | 7.2 (±4.5) | +3.0 | 0.03 | 0.08 | ns | 0.19 (±1.74) | 5.3 (±6.1) | +1.1 | 0.48 | 0.77 | ns | 0.49 (±1.08) | 4.4 (±4.0) | +0.2 | 0.88 | >0.99 | ns |
| Brainstem | 0.90 (±0.21) | 4.8 (±3.4) | 0.68 (±0.68) | 12.5 (±8.0) | +7.7 | <0.001 | <0.001 | 0.69 | 0.60 (±0.86) | 7.0 (±5.9) | +2.3 | 0.06 | 0.23 | ns | 0.70 (±0.65) | 5.6 (±4.8) | +0.8 | 0.41 | >0.99 | ns |
| DTI data - Fractional anisotropy | | | | | | | | | | | | | | | | | | | | |
| Ventricles | 0.97 (±0.07) | 4.0 (±3.0) | 0.66 (±0.72) | 10.8 (±5.3) | +6.7 | <0.001 | <0.001 | 0.87 | 0.90 (±0.22) | 5.3 (±3.7) | +1.3 | 0.12 | 0.62 | ns | 0.90 (±0.22) | 5.3 (±4.0) | +1.2 | 0.15 | 0.89 | ns |
| Supratent. WM | 0.96 (±0.10) | 1.6 (±1.4) | 0.06 (±2.01) | 4.0 (±2.7) | +2.4 | <0.001 | <0.001 | 0.64 | 0.48 (±1.11) | 2.7 (±1.8) | +1.2 | 0.004 | 0.03 | 0.34 | 0.46 (±1.17) | 2.8 (±1.9) | +1.3 | 0.002 | 0.02 | 0.38 |
| Supratent. Cortex | 0.88 (±0.25) | 1.8 (±1.4) | 0.29 (±1.53) | 4.2 (±2.9) | +2.4 | <0.001 | <0.001 | 0.61 | 0.82 (±0.39) | 1.7 (±1.4) | -0.0 | 0.92 | >0.99 | ns | 0.80 (±0.43) | 1.8 (±1.2) | +0.0 | >0.99 | >0.99 | ns |
| Supratent. deep GM | 0.90 (±0.22) | 1.2 (±1.0) | 0.75 (±0.53) | 2.2 (±1.5) | +1.0 | 0.001 | 0.003 | 0.49 | 0.92 (±0.16) | 1.0 (±0.7) | -0.2 | 0.41 | >0.99 | ns | 0.92 (±0.18) | 1.0 (±0.7) | -0.2 | 0.41 | >0.99 | ns |
| Cerebellar GM | 0.96 (±0.09) | 3.3 (±2.6) | 0.64 (±0.77) | 7.9 (±4.1) | +4.5 | <0.001 | <0.001 | 0.75 | 0.91 (±0.20) | 3.4 (±2.8) | +0.1 | 0.89 | >0.99 | ns | 0.91 (±0.18) | 3.2 (±2.9) | -0.2 | 0.79 | >0.99 | ns |
| Cerebellar WM | 0.86 (±0.30) | 5.3 (±5.2) | 0.38 (±1.33) | 6.8 (±4.8) | +1.5 | 0.22 | 0.22 | ns | 0.39 (±1.31) | 6.0 (±4.2) | +0.7 | 0.51 | >0.99 | ns | 0.38 (±1.33) | 6.9 (±4.5) | +1.6 | 0.16 | 0.89 | ns |
| Brainstem | 0.94 (±0.12) | 2.8 (±2.4) | 0.47 (±1.13) | 5.8 (±4.1) | +3.0 | 0.001 | 0.002 | 0.52 | 0.46 (±1.15) | 4.3 (±4.7) | +1.5 | 0.1 | 0.62 | ns | 0.51 (±1.04) | 3.5 (±3.7) | +0.7 | 0.35 | >0.99 | ns |

***Supplemental table 1. Scanner effect before and after harmonization for neuroCombat not using a parametric prior.*** *Healthy subjects were scanned twice less than 180 days apart on either the same scanner (Within-scanner) or on two different scanners (Across-scanner). ICC = intra-class correlation coefficient. CoV = Coefficient of variation. This can be interpreted, for example for line 1, as follows: when measuring the ventricular volume of the same subject repeatedly on the same scanner, the standard deviation across repeat scans will be 1.6% (±1.4) of the mean ventricular volume. ΔCoV is the across-scanner CoV minus the within-scanner CoV i.e., a measure of how much variation is added by using a different scanner for the second scan. The within-scanner CoV and across-scanner CoV were compared with a t-test.Raw p-value and adj. p-value are the resulting p-values before and after adjustment for multiple comparisons using Holm’s method. We considered the use of different scanners to have a significant effect if adj. p-value < 0.05. Where this was the case, the magnitude of this scanner effect is calculated as Cohen’s d with the colour coding of purple/pink/rose for large/medium/small effects with the thresholds of 0.8, 0.5 and 0.2 respectively. Non-significant scanner effects (ns) are coloured green. Supratent. = Supratentorial, WM = white matter, GM = gray matter, DTI = diffusion tensor imaging*


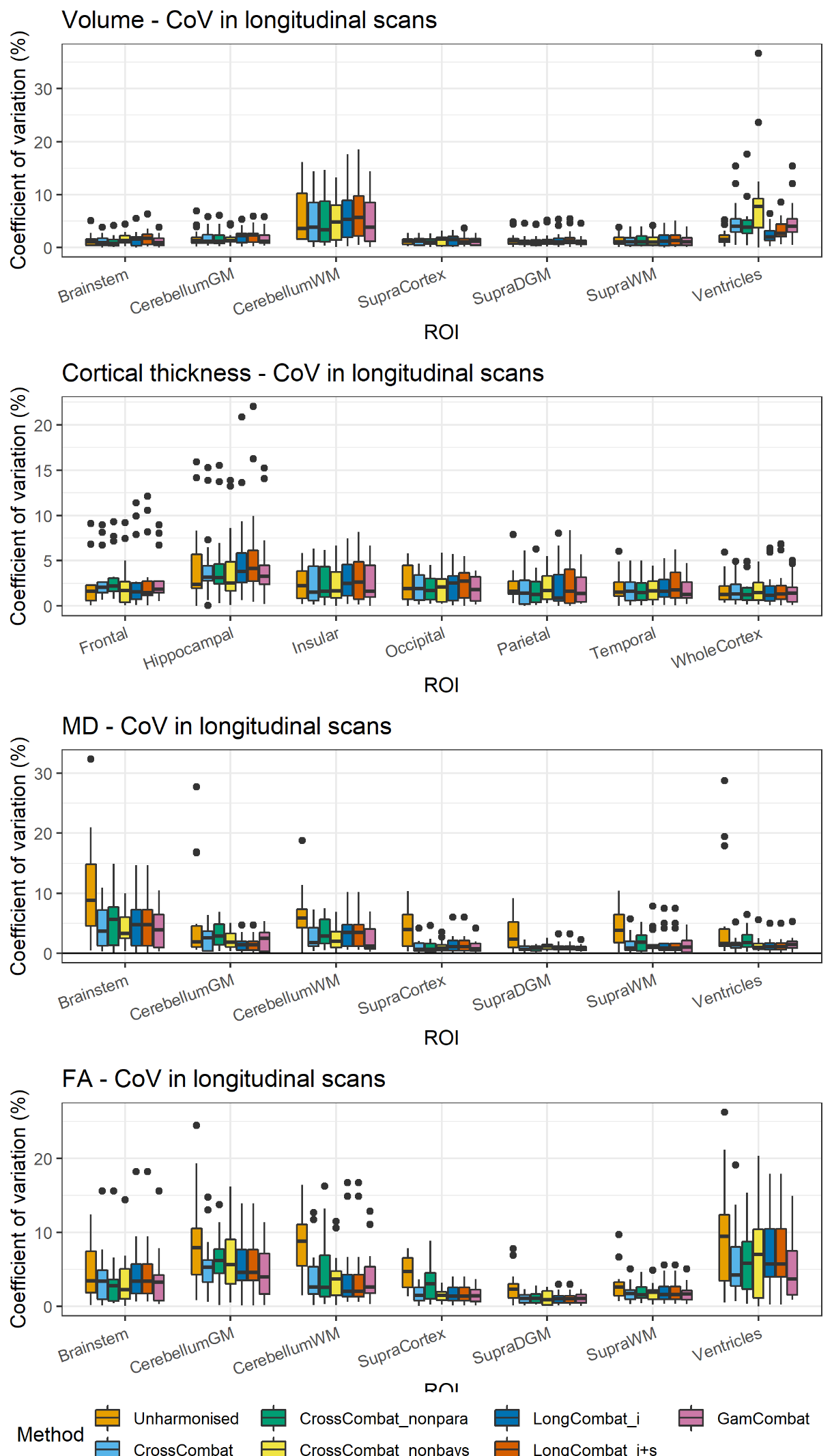


***Supplemental figure 1. Scanner effect before and after harmonization in longitudinal data.*** *Imaging metrics for the same subject were compared between two follow up scans, one on the same scanner and one on a different scanner as the initial reference scan. Boxplots show medians and interquartile ranges. CrossCombat = neuroCombat with parametric prior, CrossCombat_nonpara = neuroCombat with non-parametric prior, CrossCombat_nonbays = neuroCombat non-baysian location shift model, LongCombat = longCombat with a random intercept only, LongCombat_i+s = longCombat with a random intercept and slope for each subject, GamCombat = gamCombat assuming a non-linear effect of the covariate age during harmonization.*

*
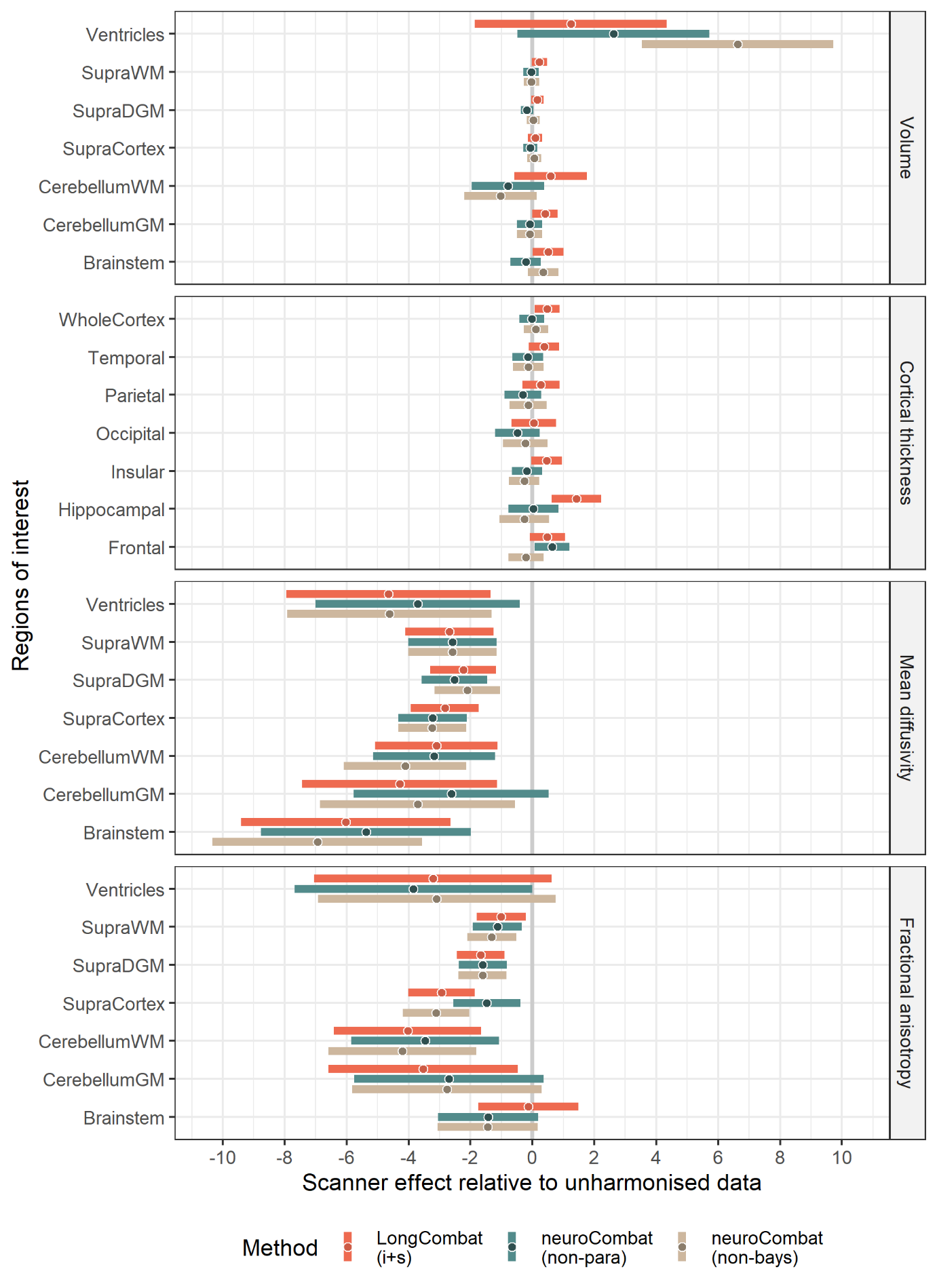
*

***Supplemental figure 2. Scanner effect relative to unharmonized data for variants of longCombat and neuroCombat methods.*** *Subjects received an initial reference scan and two follow-up scans at least one year later, one on the same and one on a different scanner. The difference between the two follow-up scans is considered to be due to differences in the hardware and acquisition settings, i.e., due to the scanner effect. The scanner effect for each subject was expressed as the percentage coefficient of variation (CoV). Here the CoV in harmonized data is shown relative to the CoV of unharmonized data, i.e., if the CoV of unharmonized data is x%, a value of -1 on the forest plot means harmonization has reduced the CoV to x-1%. Thus, points to the left of the vertical gray line (denoting zero scanner effect) indicate a reduction in scanner effect, points to the right an increase in scanner effect. A harmonization method reached statistical significance if its bars (95% confidence intervals) did not cross the gray line. The harmonization methods assessed were longCombat_i+s (longCombat with a subject-specific intercept and slope), neuroCombat (non-para) with a non-parametric prior and neuroCombat (non-bays) with a non-bayesian approach.* *Abbreviations in the names of regions of interest are: Supra = supra-tentorial, WM = white matter, (D)GM = (deep) gray matter.*
